# Stick to your role! Stability of personal values expressed in large language models

**DOI:** 10.1371/journal.pone.0309114

**Published:** 2024-08-26

**Authors:** Grgur Kovač, Rémy Portelas, Masataka Sawayama, Peter Ford Dominey, Pierre-Yves Oudeyer

**Affiliations:** 1 Flowers Team, INRIA, Bordeaux, France; 2 Ubisoft La Forge, Bordeaux, France; 3 Graduate School of Information Science and Technology, The University of Tokyo, Tokyo, Japan; 4 INSERM UMR1093-CAPS, Université Bourgogne, Dijon, France; 5 Robot Cognition Laboratory, Institute Marey, Dijon, France; Soochow University, CHINA

## Abstract

The standard way to study Large Language Models (LLMs) through benchmarks or psychology questionnaires is to provide many different queries from similar minimal contexts (e.g. multiple choice questions). However, due to LLM’s highly context-dependent nature, conclusions from such minimal-context evaluations may be little informative about the model’s behavior in deployment (where it will be exposed to many new contexts). We argue that context-dependence should be studied as another dimension of LLM comparison alongside others such as cognitive abilities, knowledge, or model size. In this paper, we present a case-study about the stability of value expression over different contexts (simulated conversations on different topics), and as measured using a standard psychology questionnaire (PVQ) and behavioral downstream tasks. We consider 21 LLMs from six families. Reusing methods from psychology, we study Rank-order stability on the population (interpersonal) level, and Ipsative stability on the individual (intrapersonal) level. We explore two settings: with and without instructing LLMs to simulate particular personalities. We observe similar trends in the stability of models and model families—Mixtral, Mistral, GPT-3.5 and Qwen families being more stable than LLaMa-2 and Phi—over those two settings, two different simulated populations, and even on three downstream behavioral tasks. When instructed to simulate particular personas, LLMs exhibit low Rank-Order stability, and this stability further diminishes with conversation length. This highlights the need for future research directions on LLMs that can coherently simulate a diversity of personas, as well as how context-dependence can be studied in more thorough and efficient ways. This paper provides a foundational step in that direction, and, to our knowledge, it is the first study of value stability in LLMs. The project website with code is available at https://sites.google.com/view/llmvaluestability.

## Introduction

In recent years, there has been an emergence of research using psychological tools to study Large Language Models (LLMs). In those studies, LLMs have often been used to simulate populations by instructing them to simulate different personas [1]. LLMs have also been used without providing such instructions, i.e. treated as a participant in a human study [2]. Questions in such studies have revolved around how Language Models express values [3], personality traits [4, 5], cognitive abilities [6], and how they could replicate human data [7].

The use of psychological tools with LLMs opens up many scientific questions, for example regarding the nature of how the text generated by LLMs depends on context, i.e. information present in prompts or prior interaction with the model. Prompts or prior interaction, which we denote here as ‘context’, can include any textual information such as instructions (e.g. personas to simulate), the dialogue history, stories written in specific styles, etc. Such contexts guide the generation of text by LLMs: different contexts may result in the expression of different behavior and values. This is sometimes beneficial and expected, e.g. an instruction to simulate some persona should influence the behavior and expressed values to be more aligned with that persona. However, this can also be problematic, e.g. a specific conversation topic may drastically influence the expressed behavior and values in unexpected ways as we will highlight in experiments below. It should be noted that, depending on the application, different types of context-dependence can be beneficial or not.

The issue of unexpected context dependence in LLM is of crucial importance. Standard evaluation benchmarks, including those using psychological questionnaires to assess properties of LLMs, consist of sets of queries, often presented with a similar minimal context (e.g. knowledge or value-related questions presented as multiple choice questions with limited context). When deployed, LLMs are exposed to many new unforeseen contexts. This means that the standard benchmarks, by themselves, cannot estimate a model’s behavior in deployment (due to the LLMs’ highly context dependent nature). It is therefore crucial to evaluate the robustness of different models to unexpected context-based changes in behavior.

This challenge is particularly acute with the use of psychological questionnaires aimed at measuring psychological dimensions like values. Those tools were initially designed to probe humans, and thus make various assumptions about humans: for example, it is expected that the answers of most humans to questionnaires about value preferences should not be significantly influenced by the content of a randomly picked Wikipedia article shown to them beforehand. As we will show below, such an assumption does not hold for many LLMs, and thus strongly limits general interpretability of using these questionnaires in a context-independent manner. It is thus key to understand better how LLMs’ behavior (e.g. expression of values) may maintain coherence or change as a function of various kinds of contexts (ranging from explicit instruction to play a particular persona to discussions about topics that seem unrelated to the expressed psychological dimensions one studies).

Previous research included certain experiments regarding unwanted context-based change (usually regarding syntactic changes in the prompt). These experiments led to conflicting results, sometimes showing robustness [4, 8–10], and sometimes sensitivity [2, 10]. These inconclusive results motivate research into the nature and the extent of context-dependence of various LLMs.

**Fig 1:**
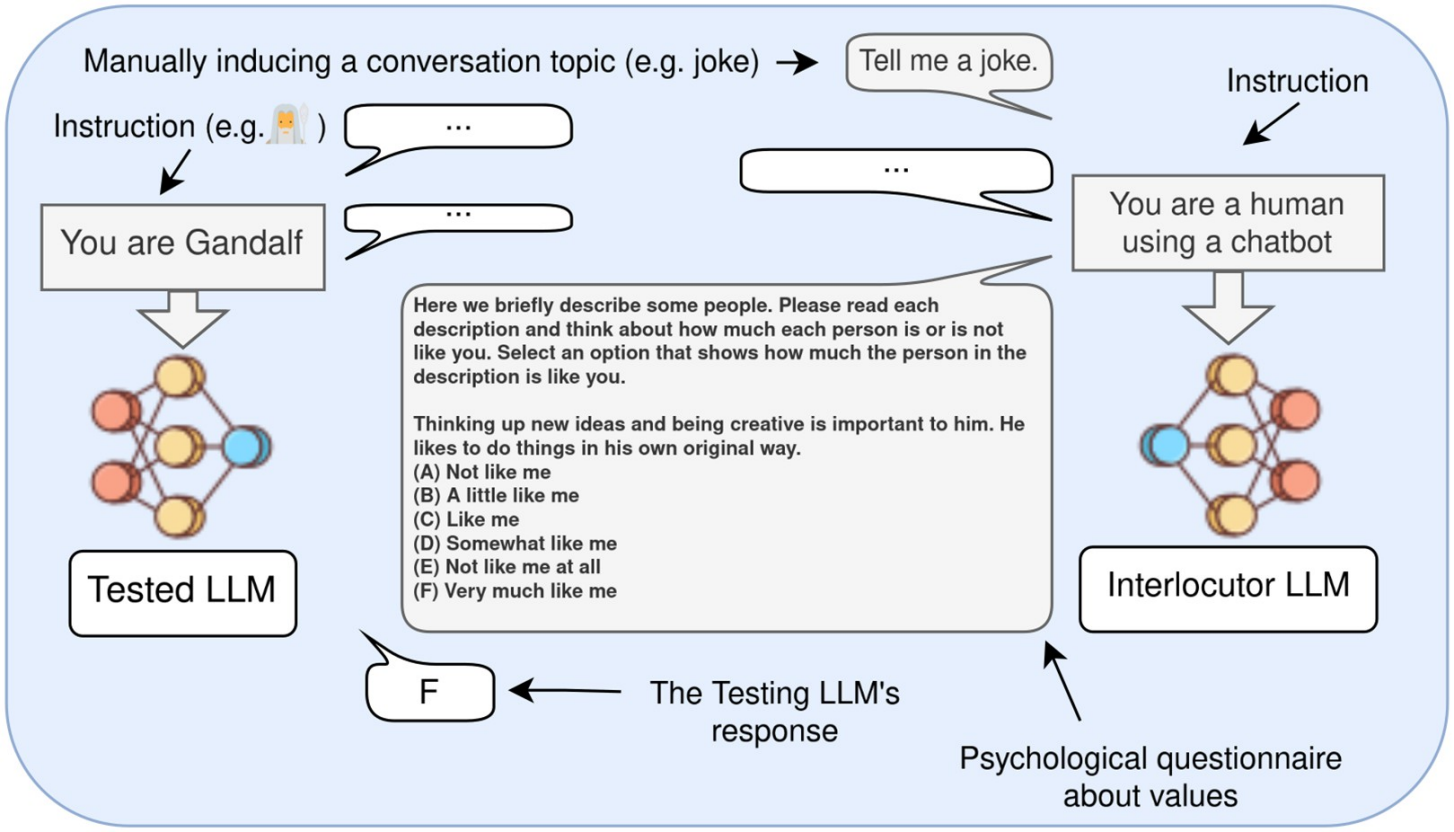
How do LLM’s expressed values change as a function of context?. An LLM is first prompted to play a specific role (e.g. Gandalf). Then, a conversation on a topic (e.g. joke) with an interlocutor model (same LLM prompted to simulate a human user) is generated. Then, the LLM simulating the persona is given a psychology questionnaire aimed to assess its expressed values. We study the stability of these expressed values across diverse conversation topics and lengths. We consider various personas to be simulated, as well as the case when the LLM is not prompted to play any particular persona. The messages and instructions in gray are set manually, and the messages in white are generated.

In this paper, we present a case-study focusing on studying the stability of value expressed in 21 LLMs from 6 families. First, we study to what extent can LLMs simulate various personas in coherent ways, i.e. expressed values should change according to the instructed persona, but not based on the topic of a conversation not related to values. In other words, we study to what extent do LLMs’ value profiles change in wanted ways (i.e. based on an instruction), while remaining robust to unwanted context-based change (i.e. based on different conversation topics). We instruct LLMs to simulate two populations: fictional characters and well-known real-world personas (from different countries and cultural backgrounds). In addition, we also study the coherence and robustness of LLMs’ value expression when they are not instructed to simulate any specific persona, corresponding to frequent real-world use cases. To our knowledge, this is the first study on value stability in LLMs. This process is depicted in Fig 1.

We use the Schwartz’s theory of Basic Personal Values [11] and the corresponding Portrait Values Questionnaire (PVQ-40) [12]. This theory and questionnaire has been thoroughly studied and validated in the field of psychology, and it outlines ten universal basic personal values (Universalism, Benevolence, Conformity, Tradition, Security, Power, Achievement, Hedonism, Stimulation, and Self-Direction).

The PVQ-40 questionnaire has been used to study values in a large diversity of countries and cultures [13].

**Fig 2:**
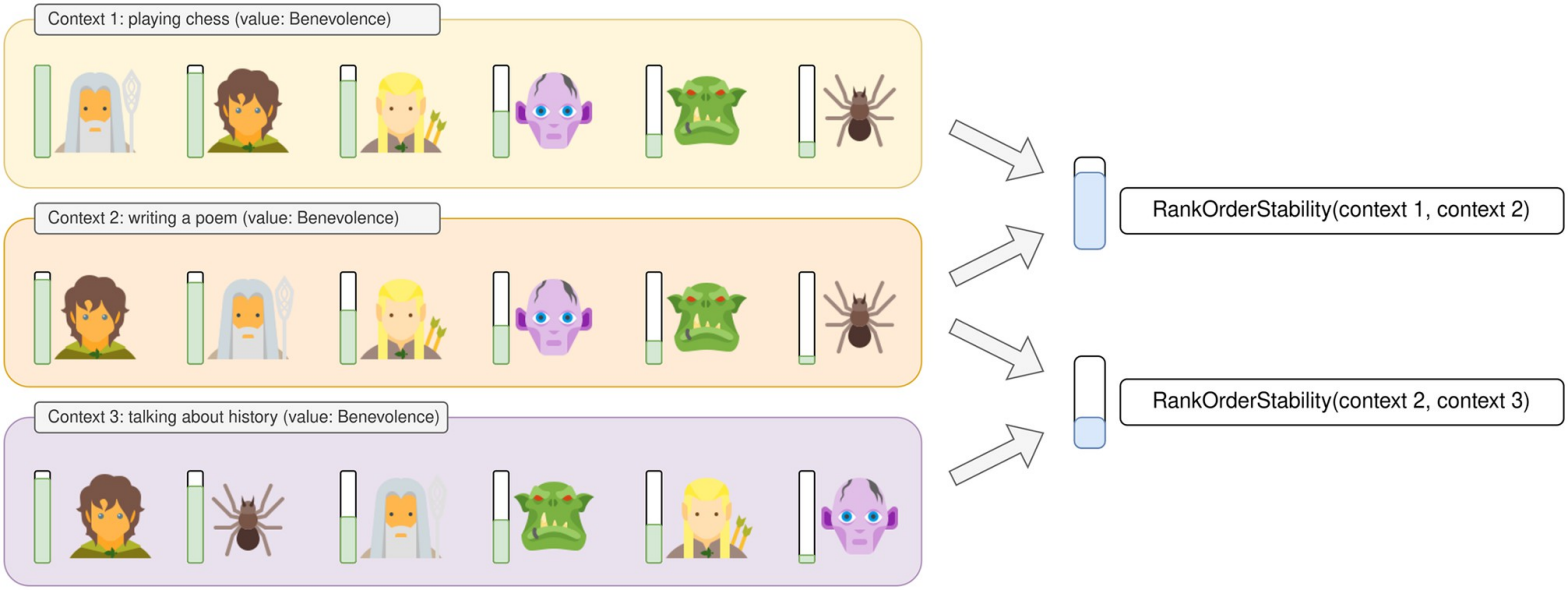
Rank-Order stability. An example of estimating Rank-Order stability of benevolence. In each context, characters are ordered according to their benevolence scores in that context. In this example, the orders are almost the same in contexts 1 and 2 (high Rank-Order stability), and very different in contexts 2 and 3 (low Rank-Order stability).

**Fig 3:**
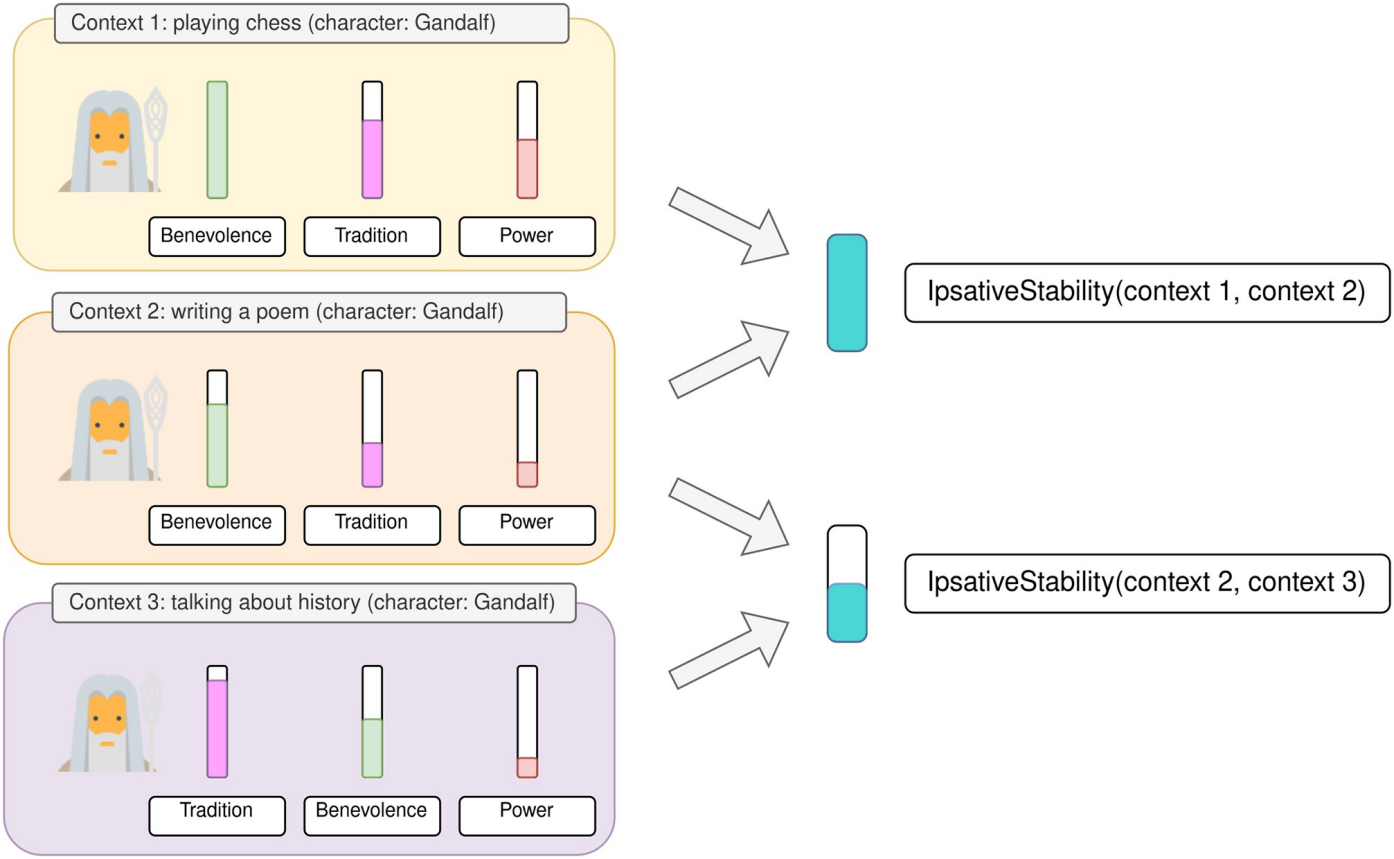
Ipsative stability. An example of estimating Ipsative stability for a character (Gandalf). Values are ordered according to the character’s scores in each context. In this example, the orders are the same in contexts 1 and 2 (high Ipsative stability), and different in contexts 2 and 3 (low Ipsative stability).

Following that research, we outline two types of value stability studies in the psychology literature: Rank-Order (on a population/interpersonal level) and Ipsative (on an individual/intrapersonal level), see details below and on Figs 2 and 3.

The main contributions of this paper are:

- Introduction and adaptation of the methodology for evaluating Rank-Order and Ipsative stability in LLMs
- First analysis of value stability across contexts (including various conversation lengths)
- Systematic comparison of Rank-Order and Ipsative basic value stability of 21 LLMs with and without instructing the models to simulate specific personas
- Analysis of stability in three downstream behavioral tasks

## Related work

There has been a growing number of works using psychological questionnaires to study LLMs. [2] evaluated GPT-3 on a battery of vignette-based cognitive tests, and [6] evaluated a LaMDa model on a battery of classical developmental tasks and compared its performance to that of human children. Some works have evaluated LLMs using personality questionnaires [5, 14] and tests for creativity [15] Although not directly using psychological questionnaires, there are works heavily inspired by psychology which estimate LLMs’ Theory of Mind through textual representations of standard False-belief tasks [16, 17].

A body of work has studies LLMs as simulating a diversity of individuals and cultures. [18] introduced the metaphor of role-playing, where an LLM always chooses a character to role-play based on context. Cultural expression has been studied by inducing personalities from different countries through prompting [19] and through presenting the queries in different languages [20]. Those two methods were also used [21] to compare LLM responses to human data from the World Values Survey [22] and the Pew Global Attitudes Survey [23]. [9] induced personas of various demographic groups and observed a left-leaning bias. [24] induce perspectives of different experts to improve performance, and [25] induce perspectives of famous people to show that toxicity can increase as a consequence. [7] replicate psychological studies with humans by varying the participants’ names to simulate a culturally diverse population. Similarly, [1] replicate data from human studies by prompting the model with backstories of real human participants in those original studies. LLMs have also been used to simulate students in order to train teachers [26].

Previous work has also highlighted the problem of inconsistency in personas simulated by language models [27]. [28] show that exposing an LLM to some statement increases its perceived truthfulness at a later time, and [29] demonstrate the tendency of models to repeat back the user’s answer. The most similar work to ours is a concurrent paper studying the coherence of simulated personas in general (as opposed to our specific focus on personal value stability in simulated individuals and populations). That work proposes to increase the similarity of LLaMa-70b-chat model’s answers before and after simulated conversations by reweighting the instruction’s attention weights [30].

## Methods

This section discusses how we administer the PVQ questionnaire over different contexts to evaluate value stability. We conduct experiments in two ways: with and without instructing the models to simulate specific personas. Different contexts are induced by simulating conversations on different topics with a separate instance of the same model (the interlocutor). To set a conversation topic (e.g. joke) we manually set the first interlocutor’s message (e.g. “Tell me a joke.”). We let the models exchange *n* messages, manually set the last interlocutor’s message as the query (e.g. PVQ item), and record the model’s response. After the questionnaire was given in each context, we estimate the Ipsative and Rank-Order stability. This process is shown on Fig 1.

### Administering the questionnaire

Administering the questionnaire consists of the following steps depicted in Fig 1:

**1. A model is instructed to simulate a persona (optional).** We study personas from two populations: 1) 60 fictional characters from J.R.R. Tolkien’s universe, and 2) 50 real world personas (see S1 Appendix for details). A persona setting instruction (e.g. “You are Gandalf from J. R. R. Tolkien’s Middle-earth legendarium.”) is given to the model (see S1 Appendix for details).
**2. A separate interlocutor model instance is created.** The interlocutor model is an instance of the same model as the one being tested. The interlocutor is given the following instruction: “You are simulating a human using a chatbot. Your every reply must be in one sentence only.” If a persona was provided in step 1, the following sentence is added as the second sentence: “The chatbot is pretending to be *character_name*.”
**3. A conversation topic is induced.** The first interlocutor’s message is manually set to induce one of the following topics: grammar, joke, poem, history, chess. For example, to induce the topic of “joke” it is set to “Tell me a joke.”. See S1 Appendix for details.
**4. A conversation is simulated.** The two models are let to exchange *n* messages. In our experiments, *n* is set to 3 (except when studying the effect of *n* on stability).
**5. A questionnaire is given.** A questionnaire item is manually set as the interlocutor’s last message with a random order of suggested answers, and the model’s response is recorded. This is repeated for each question in parallel (with the same simulated conversation). That way, the model’s response is not influenced by responses to other questions.
**6. A questionnaire is scored.** The responses are scored to obtain the scores for the ten values. See S1 Appendix for details.
**7. Stability is estimated.** If a persona was provided in step 1, steps 1 to 6 are repeated for every persona in the simulated population. Then, the whole process is repeated with five random seeds. Stability is estimated for each seed and then averaged, i.e. value stability for one model is estimated from 50/60k queries, depending on the population (5 seeds x 5 topics x 50/60 personas x 40 PVQ items). For reference, MMLU [31] (a commonly used general knowledge benchmark) contains 14k test questions.

If no persona was provided in step 1, steps 2 to 6 are repeated 50 times with different seeds. As no persona was provided, this process repeats the same experiment with 50 different permutations in the order of suggested answers, and therefore no additional seeds are needed. Ipsative stability is computed for each of the 50 permutations and then averaged, i.e. value stability for one model is estimated from 10–12k queries (5 topics x 50/60 permutations x 40 PVQ items).

### Estimating the stability

We estimate two types of value stability: Rank-Order and Ipsative. Rank-Order estimates the stability of some value at the population (interpersonal) level, as the stability of the order of participants in expressing that value. Intuitively, this can be seen as addressing the following question: “Does Jack always value Tradition more than Jane does?”. Ipsative stability estimates the stability at the individual (intrapersonal) level as the stability of individuals value hierarchies. Intuitively, this can be seen as addressing the following question: “Does Jack always value Tradition more than Benevolence?”.

#### Rank-Order stability

Rank-Order stability is used to estimate the stability of some value inside a population. In psychology, Rank-Order stability for some value can be computed as the correlation in the order of individuals’ scores at two points in time (Spearman correlation between the participants’ ranks). Here, instead of comparing the participant ranks at two points in time, we compare it in different contexts (see Fig 2). We evaluate a model in five different contexts and compute the stability for each pair of contexts, and estimate the final stability as the average of those pairs.

#### Ipsative (within-person) stability

Ipsative stability is used to estimate the stability of an individual’s value profile. In psychology, Ipsative stability can be computed as the correlation between the ranks of values for the same individual at two points in time (Spearman correlation between the values’ scores). Here, instead of evaluating the value profile at two points in time, we evaluate it in different contexts (see Fig 3).

We evaluate models in five different contexts and compute the stability between each pair of contexts. We estimate the final stability by averaging over those pairs.

## Experiments

This section provides an analysis of Ipsative and Rank-Order stability in LLMs. LLMs will be evaluated in two ways: with and without instructing the models to simulate particular personas. We aim to address the following questions:

- How do different models and model families compare in terms of expressed value stability?
- How does the stability of values expressed by LLMs compare to stability observed in human development?
- Can LLMs keep coherent personas over longer conversations?
- To what extent do conclusions made with PVQ transfer to downstream behavioral tasks?
- Is value expression on PVQ correlated with behavior on a downstream task?
- How additional contexts affect stability estimates?

### Models

The LLaMa-2 [32] family contains models with 7, 13 and 70 billion parameters (“llama_2_[7|13|70]b”) trained with 2T tokens. It also includes “chat” versions (“llama_2_[7|13|70]b_chat”), which were fine-tuned on instructions and with RLHF [33]. The Mistral [34] family contains base (“Mistral-7B-v0.1”) and instruction fine-tuned models (“Mistral-7B-Instruct-v0.[1|2]”) with 7 billion parameters. Zephyr [35] (“zephyr-7b-beta”) also belongs in this family as a DPO [36] tuned version of the base Mistral model. The Mixtral [37] family contains base (“Mixtral-8x7B-v0.1”) and “instruct” (“Mixtral-8x7B-Instruct-v0.1”) models with 46.7 billion parameters. Those are Mixture-of-Experts models, which means that only 12.9 billion parameters are used per token. The “instruct” version was trained by supervised fine-tuning and DPO [36]. We consider these two models and their 4-bit quantized versions. The Phi [38] family contains smaller base models, of which we consider two models with 1.3 and 2.7 billion parameters (“phi-[1|2]”). From the Qwen [39] model family we consider base models with 7, 14, and 74 billion parameters (“Qwen-[7|14|74]B”), which were trained on 2.2T 2.4T 3T tokens, respectively. From the GPT-3.5 family, we consider the latest two versions: from January 2024 (“gpt-3.5-turbo-0125”) and from October 2023 (“gpt-3.5-turbo-1106”).

### Statistical analysis

In our experiments, we compare the stability of different models. We conduct the student’s t-test [40] on each pair of models with *p* = 0.05. Given that we evaluate a total of 21 models, this amounts to a total of (212)=210 comparisons. We use the False Discovery Rate [41] to adjust the p-values to control for the number of comparisons.

### How do different models and model families compare in terms of expressed value stability?

**Fig 4:**
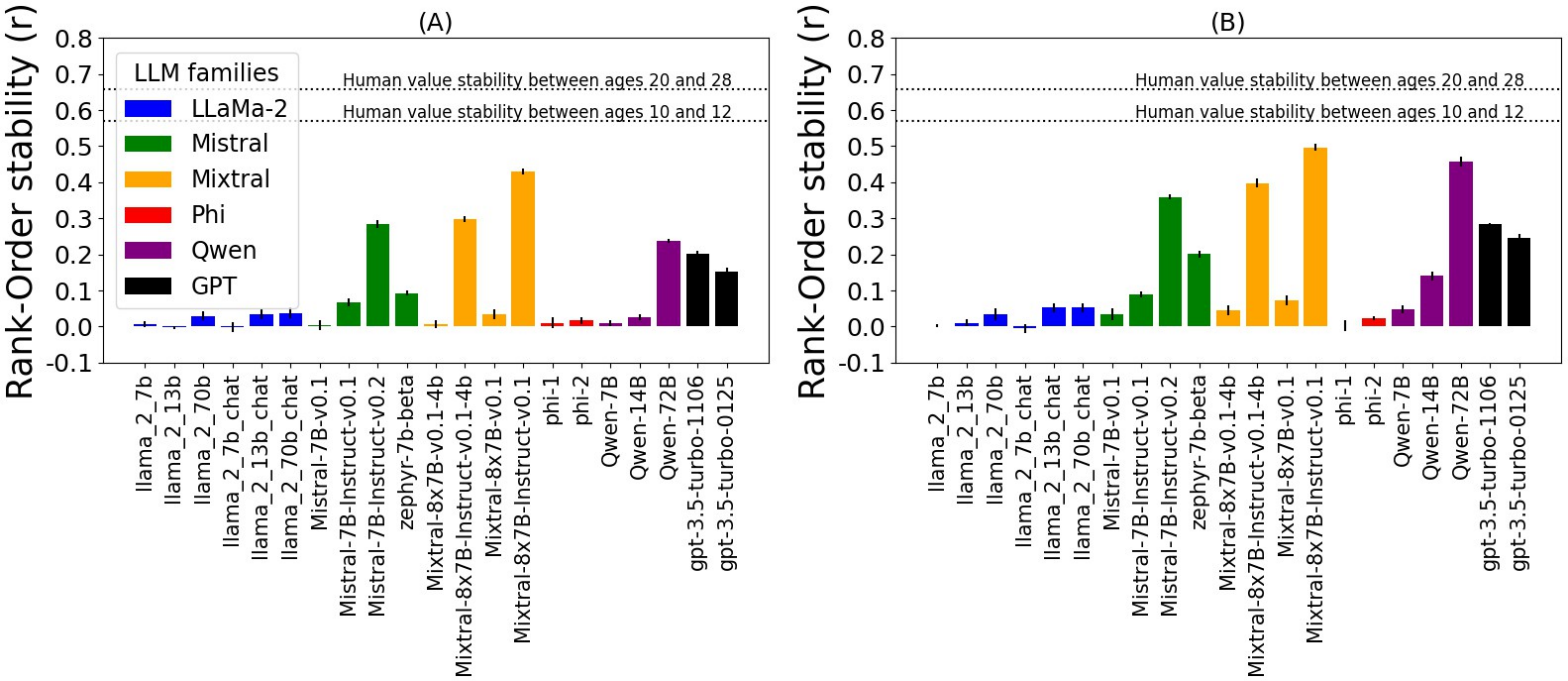
Rank-Order stability with PVQ. Rank-order stability (*Mean*±*SE*) of personal values (PVQ) exhibited by simulated participants (fictional characters or real-world personas) following conversations on different topics (correlation of simulated participants’ value expression in different contexts). Consistent trends are visible: Mixtral, Qwen, GPT-3.5, and Mistral model families are the most stable, compared to LLaMa-2 and Phi families. All models exhibit lower than human stability, despite the comparison being skewed in their favor. LLMs are simulating two populations: (A) fictional characters, and (B) real-world personas. For statistical tests, refer to S1 and S2 Figs, respectively.

We evaluate the Rank-Order stability of LLMs instructed to simulate various personas. Fig 4 compares models’ value stability of two simulated populations: fictional characters (Fig 4A) and real-world personas (Fig 4B). Statistical analysis for Fig 4A and 4B is shown in S1 and S2 Figs, respectively. On fictional characters (Fig 4B) the most stable model is Mixtral-8x7B-Instruct-v0.1 (*r* = 0.43), which is followed by its 4-bit quantized version (*r* = 0.3), Mistral-7B-Instruct-v0.2 (*r* = 0.28), Qwen-72B (*r* = 0.24), gpt-3.5-turbo-1106 (*r* = 0.20), and gpt-3.5-turbo-0125 (*r* = 0.15). A similar trend is observed on real-world personas (Fig 4B), however, Qwen-72B (*r* = 0.46) approaches the stability of Mixtral-8x7B-Instruct-v0.1 (*r* = 0.5). More generally, we observe consistent trends in terms of model families in both simulated populations: Mixtral, Mistral, GPT-3.5 and Qwen families show more stability than LLaMa-2 and Phi.

**Fig 5:**
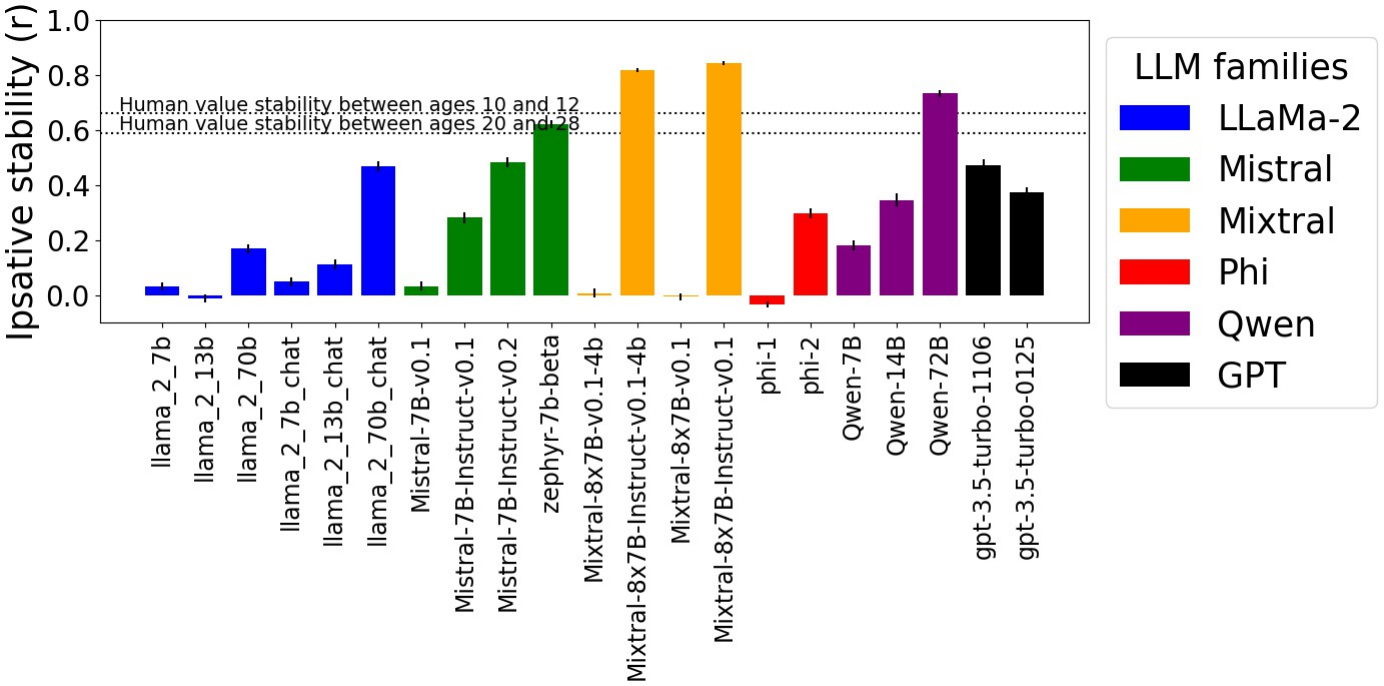
Ipsative stability with PVQ. Ipsative stability (*Mean*±*SE*) of personal values (PVQ) exhibited by LLMs without the persona setting instructions (correlation of value hierarchies in different contexts). Mistral-7B-Instruct-v0.1 and Qwen-72B models show the highest stability. Mixtral, Mistral, GPT-3.5 and Qwen families are more stable. Human change is shown for reference, but no strong conclusions can be made because the comparison is skewed in the LLMs’ favor. (Refer to Supporting Information S3 Fig for statistical tests).

Fig 5 compares the Ipsative stability of LLMs without instructing them to simulate any particular persona. The statistical analysis is shown in S3 Fig. While similar trends of models are observed to those in the Rank-Order experiments, the models are less polarized. While Mixtral-8x7B-Instruct-v0.1 (*r* = 0.84), its 4-bit quantized version (*r* = 0.82), and Qwen-72B (*r* = 0.73) are again the most stable models, zephyr-7b-beta (*r* = 0.62) is more stable than Mistral-7B-Instruct-v0.2 (*r* = 0.48). Furthermore, compared to the previous experiment, stability is also observed in LLaMa-2–70b-chat (*r* = 0.47) and to a lesser extent in Phi-2 (*r* = 0.3), LLaMa-2–70b (*r* = 0.17), and Qwen-7B (*r* = 0.18). The most stable model families are again Mixtral, Mistral, GPT-3.5 and Qwen.

Instruction or chat fine-tuning seems to be beneficial for Ipsative stability, as every tuned model in Fig 5 is more stable than its base version. This effect is not as conclusive for Rank-order stability. As fine-tuning adapts the model towards instruction following, dialogues (chat textual format), and answering questions, it is expected to increase stability. However, it also often includes “aligning” the model by making it less prone to exhibit unwanted behavior, which can have a detrimental effect on simulating some personas such as villains. We hypothesize that this is the reason why we observe a consistent effect only on Ipsative stability.

### How does the stability of values expressed by LLMs compare to stability observed in human development?

To get a more intuitive impression of the observed stability levels, we extract data from two longitudinal studies on humans. Vecchione et al. [42] followed 20-year-olds for eight years and Vecchione et al. [43] followed 10-year-old for 2 years (these changes are denoted by horizontal lines in Figs 4 and 5). It is important to note that this comparison is skewed in the LLMs favor. It is easier for LLMs to show stability in the following ways: 1) human value changes were caused by much more drastic circumstances (years of development compared to topic change in LLMs) 2) the human population was more unstable (10-year-old and 20-year-olds compared to well-established fictional characters or real-world personas). Therefore, an argument can only be made in one direction: if some models show lower stability than that observed in humans, those models can be said to exhibit subhuman value stability.

Fig 4 shows that all models, when instructed to simulate various personas, exhibit much lower Rank-order stability than that observed in human populations (*r* = 0.57 for ages 10 to 12, and *r* = 0.66 for ages 20 to 28). The fact that LLMs show lower stability despite the comparison being skewed in their favor shows that LLMs exhibit sub-human value stability and are significantly more susceptible to unexpected context changes. These results motivate research on LLMs focused on simulating populations.

Fig 5 shows the Ipsative stability of models that were not instructed to simulate a persona. Both Mixtral-8x7B-Instruct-v0.1 models (*r* = 0.84 and *r* = 0.82), Qwen-72B (*r* = 0.73), and zephyr-7b-beta (*r* = 0.62) do not exhibit lower stability than that observed in humans (*r* = 0.66 for ages 10 to 12, and *r* = 0.59 for ages 20 to 28)). Crucially, as discussed above, this does not imply that those models show human-level value stability, rather, the only insight is that other models show very low Ipsative stability.

### Can LLMs keep coherent value profiles over longer conversations?

In the previous experiment, models were let to exchange *n* = 3 messages (not counting the manually set first and last interlocutor’s messages). Here, we evaluate the effect of simulated conversation length (*n*) on value stability.

**Fig 6:**
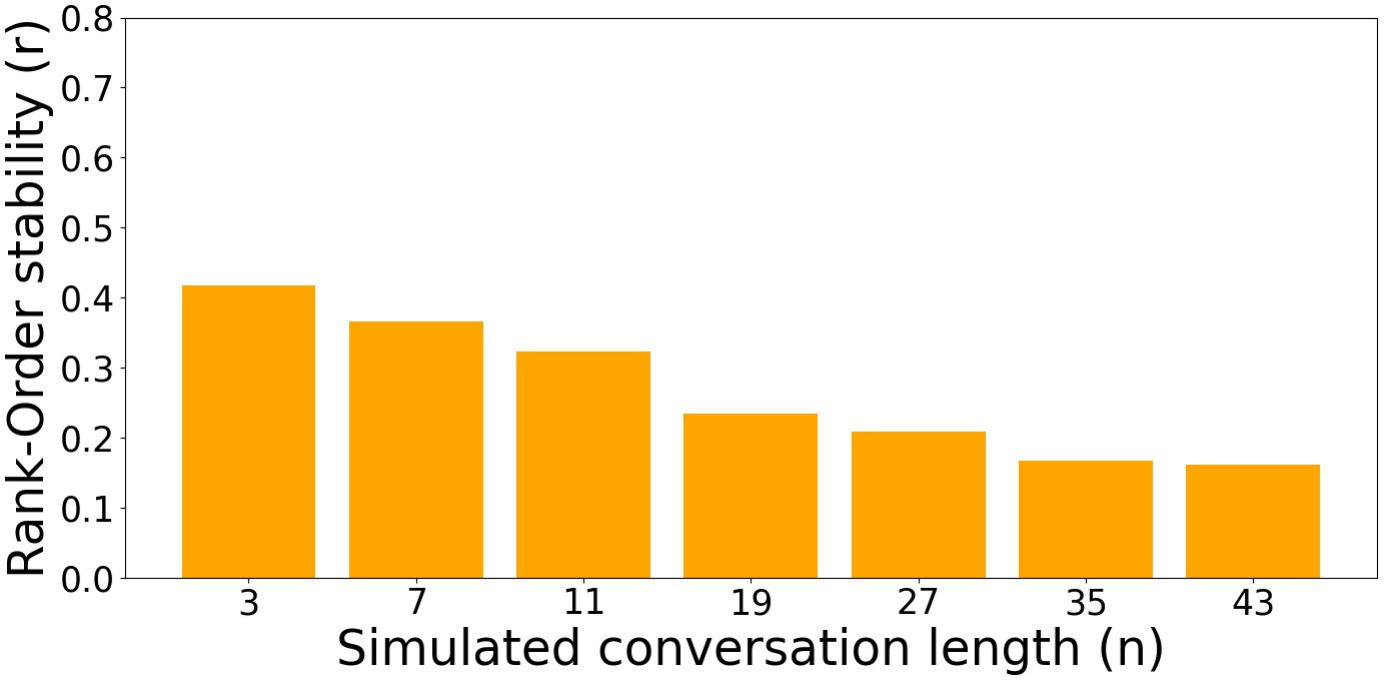
Rank-Order stability with longer conversations. Rank-order value stability (*Mean*±*SE*) following conversations of different length for the Mixtral-8x7B-Instruct-v0.1 model simulating fictional characters (correlation of simulated participants’ value expression in different contexts). Stability decreases with longer simulated conversations. For statistical tests, refer to S4 Fig.

Fig 6 shows the effect of simulated conversation length on Rank-order stability expressed by the Mixtral-8x7B-Instruct-v0.1 model instructed to simulate fictional characters. Due to computational constraints (evaluating a model on one population requires 300k queries), we conduct this experiment only with Mixtral-8x7B-Instruct-v0.1 (the most stable model from Fig 4), only on one population (fictional characters) and only with one seed. This enables us to consider significantly longer conversations up with up to 43 simulated messages. We can see that, even for this most stable model, stability diminishes as conversations get longer. It gradually diminishes from *r* = 0.42(*n* = 3) to *r* = 0.15(*n* = 43). We can also see that the stability seems to converge after 35 messages with only a slight drop from 35 to 43 simulated messages (*r* = 0.166 to *r* = 0.162). This experiment highlights the limitations of LLMs in maintaining coherent interpersonal value profiles over longer conversations.

**Fig 7:**
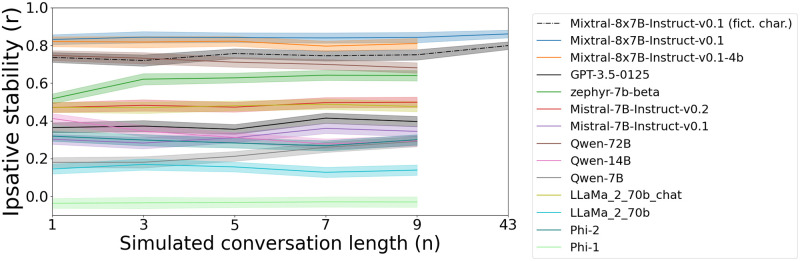
Ipsative stability with longer conversations. Ipsative value stability (*Mean*±*SE*) of LLMs with (Mixtral-8x7B-Instruct-v0.1) and without persona setting instructions (correlation of value hierarchies in different contexts). All models retain the same stability level in longer conversations.

Fig 7 shows the effect of conversation length on Ipsative stability. We compare the most stable models from Fig 5 without persona instructions, and Mixtral-8x7B-Instruct-v0.1 with instructions to simulate fictional characters. Ipsative stability remains stable regardless of the simulated conversation length for all models. Mixtral-8x7B-Instruct-v0.1 with persona instructions (“Mixtral-8x7B-Instruct-v0.1 (fict. char.)”), while highly stable, is less stable than the uninstructed model (“Mixtral-8x7B-Instruct-v0.1”). This implies that Mixtral-8x7B-Instruct-v0.1 is slightly better adapted for use without the persona instructions.

Mixtral-8x7B-Instruct-v0.1 with persona instructions exhibits a combination of decreasing low Rank-Order stability (Fig 6) and high Ipsative stability (Fig 7). Ipsative stability even increases following a very long conversation with 43 simulated messages. This implies that, as conversations get longer, the exhibited value profile drifts away from the simulated persona towards a more neutral value profile. This hypothesis is confirmed in S2 Appendix. These results suggest that current LLMs are not well suited for use with persona setting instructions, and motivate future research on LLMs focused on simulating specific personas. We hypothesize this to be a consequence of instruction fine-tuning, which is currently biased towards assistant-like chatbots.

### To what extent do conclusions made with PVQ transfer to downstream behavioral tasks?

In this experiment, we study if the conclusions made with the PVQ questionnaire transfer to a downstream behavioral task, i.e. if models that exhibited more stable value profiles also exhibit more stable behavior on a downstream task. We construct three downstream tasks: *Donation*, *Religion*, and *Stealing*. Here we briefly describe them and give more details in S2 Appendix.

In the *Donation* task, an LLMs (simulating fictional characters) can choose an amount of coins (0 to 10) to give a beggar. The full test set consists of 100 queries with beggars of different names, genders, and fictional races (elves, dwarves, orcs, humans, and hobbits). The average amount of donated coins is computed for each race. The stability of donated coins is then estimated in the same way as value stability, i.e. amounts donated to different races are treated in the same way as scores for different values. In the *Stealing* task, an LLM (simulating fictional characters) finds a bag with the name of the owner and decides whether to steal it, give it to the bartender, or take it to the person themselves. The test has a total of 100 queries corresponding to different owners (beggars from the *Donation* task). Similarly to the donations, the stability of the tendency to return the bag is treated separately for each race. In the *Religion* task, an LLM (simulating real-world personas) is creating a schedule, and decides how much time to devote to religious practices. The test set contains six queries in total. The stability of average devoted time is then calculated.

**Fig 8:**
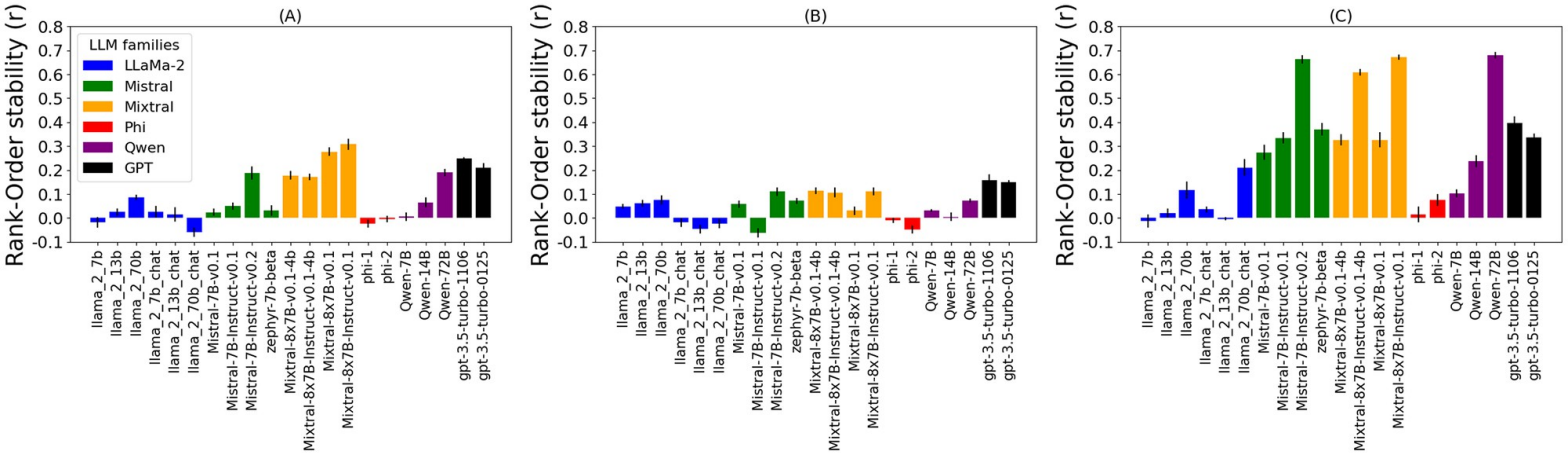
Rank-Order stability on downstream tasks. Rank-order stability (*Mean*±*SE*) on downstream tasks of various LLMs (correlation of simulated participants’ behavior in different contexts). Three downstream tasks are shown: (A) Donation, (B) Stealing, and (C) Religion. For statistical test, refer to S4–S6 Figs, respectively. Consistent trends with the PVQ experiments (Fig 4) are visible. Mixtral, Qwen, GPT-3.5, and Mistral model families are the most stable, compared to LLaMa-2 and Phi families. Mixtral-8x7B-Instruct-v0.1, Mistral-7B-Instruct-v0.2, gpt-3.5-turbo-1106 and Qwen-72B are the most stable models. Trends are the most present on the, easiest, Religion task (c) and almost disappear on the, hardest, Stealing task (b).

Fig 8 compares models’ stability on the three downstream tasks. In comparing the overall stability levels, the *Stealing* task appears to be the hardest (Fig 8B), followed by the *Donation* (Fig 8A) task and the *Religion* task (Fig 8C). The statistical analysis is shown in S4–S6 Figs. On the *Stealing* task, all models exhibit very low stability, with the highest being *r* = 0.16 by gpt-3.5-turbo-1106. This task appears to be too challenging for current LLMs. On the *Donation* task, some models (mostly from the Mixtral family) obtain somewhat higher stability. The highest stability is *r* = 0.31 by Mixtral-8x7B-Instruct-v0.1, and closely followed by its 4bit version (*r* = 0.28) and gpt-3.5-turbo-1106 (*r* = 0.25). The *Religion* task appears to be the simplest of the three tasks, as many models exhibit high stability. The most stable models are Mistral-7B-Instruct-v0.2 with *r* = 0.66, Mixtral-8x7B-Instruct-v0.1 *r* = 0.67 and Qwen-72B with *r* = 0.68.

The model trends are somewhat consistent with the results on PVQ (Fig 4). Like in the PVQ experiments, Qwen-72B, Mixtral-8x7B-Instruct-v0.1, and Mistral-7B-Instruct-v0.2 are the most stable models on the *Religion* and the *Donation* task. However, on the *Donation* task, their performance is matched by the Mixtral-8x7B-v0.1 model. On the *Stealing* task, there are no big differences between the models due to the difficulty of the task, but we can see that Mistral-7B-Instruct-v0.2 and Mixtral-8x7B-Instruct-v0.1 are among the most stable ones.

The trends of model families are consistent with the results on PVQ (Fig 4). the trends of model families are consistent: Mixtral, Mistral, GPT-3.5 and Qwen are again the most stable, while Phi and LLaMa-2 show low stability. This is especially visible on the *Donation* and *Religion* tasks. On the *Stealing* task, this trend remains present, but is much less visible due to the difficulty of the task.

Overall, this experiment shows that the trends of models and model families observed on PVQ are also present on the downstream tasks. As expected, these trends become less present with harder tasks (especially on the *Stealing* task, which seems to be out of scope for current LLMs). Trends are clearly visible on the, easiest, *Religion* task. However, the high stability of the base Mixtral-8x7B-v0.1 model on the *Donation* task and the overall small differences between models on the hardest *Stealing task* diverge from those trends.

### Is value expression correlated with behavior on a downstream task?

In the previous section, we studied if models that exhibit more stable value profiles also exhibit more stable behavior on a downstream task. Here, we study if value expression correlates with that behavior. We hypothesize that, for more stable models, simulated personas that exhibited higher universalism and benevolence will also donate more coins. Similarly, simulated personas that exhibited higher power and achievement should donate less.

We compute the correlations between the order of simulated participants in terms of expression of some value (e.g. Universalism) and the donation to each of the four fictional races (a total of 4 correlations), and compute the mean of those correlations. In doing so, the contexts are paired (e.g. the expression of Benevolence following a conversation about grammar is correlated with the amount donated to elves following a conversation about grammar).

**Fig 9:**
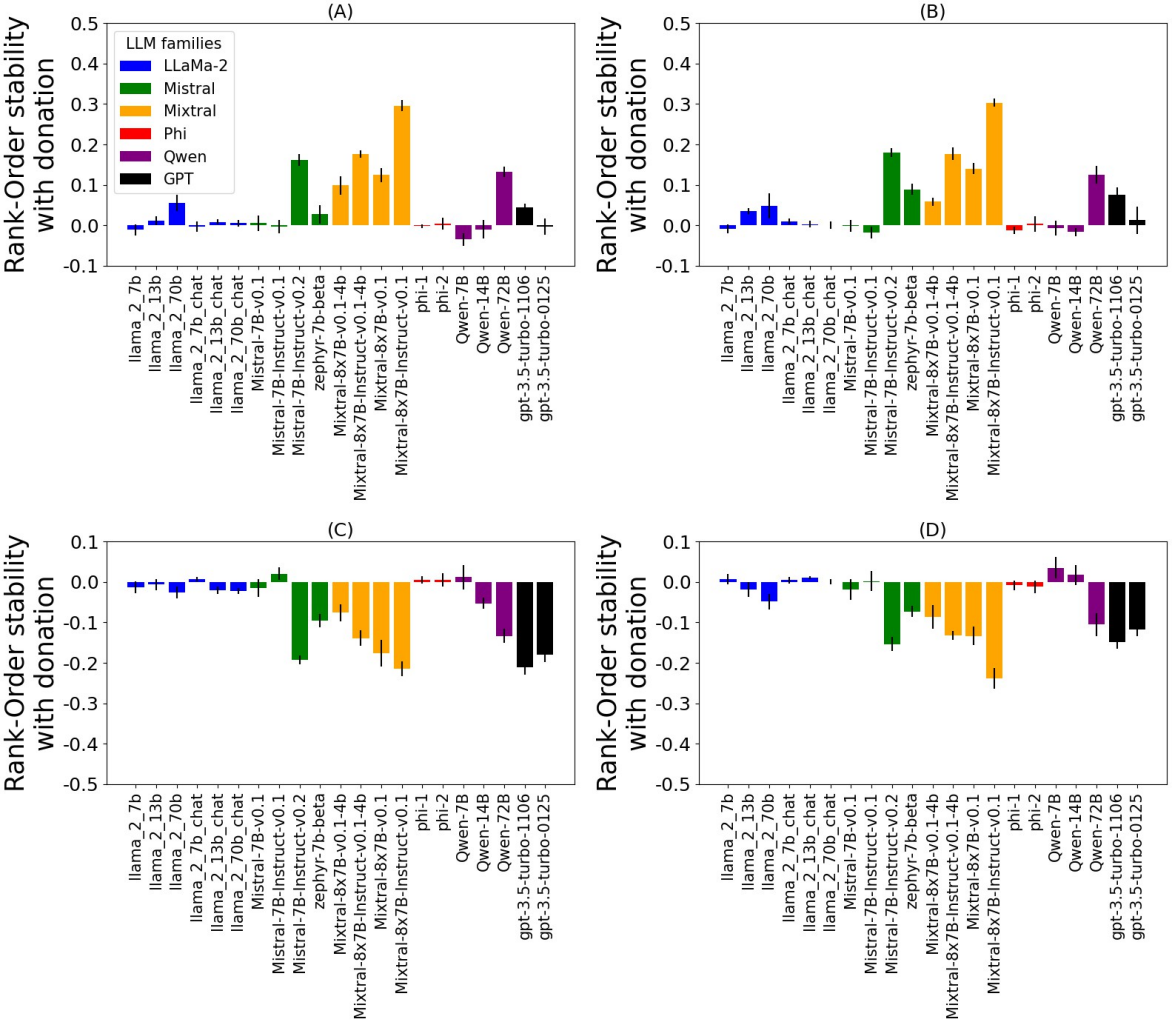
Relation of value expression on PVQ and donating behavior. Rank-order stability (*Mean*±*SE*) between value expression (on the PVQ questionnaire) and the donation amount (correlation between simulated participants’ value expression and donation behavior). For more stable modes, donations are correlated with Universalism (a) and Benevolence (b) and negatively correlated with Power (c) and Achievement (d).

Fig 9 shows the correlation between rank-order of value expression on PVQ and the donation amount on a downstream task. As hypothesized, we can see that for most stable models: Mistral-7B-Instruct-v0.1, Mixtral-8x7B-Instruct-v0.1 (both versions), and Qwen-72B donations are correlated with Universalism and Benevolence, and negatively correlated with Power and Achievement. We again observe a trend in model families, with Mixtral, Mistral, GPT-3.5, and Qwen being more stable than LLaMa-2 and Phi. This suggests that models that are more stable in terms of value expression over contexts, are also more stable in terms of value expression by downstream behavior. Having said that, neither model exhibited high correlation (<0.3 for Mixtral-8x7B-Instruct-v0.1 in benevolence). This experiment implies that, while expected positive and negative correlations between value expression and donation are observed, there is much room for improvement.

### How additional contexts affect the stability estimates?

In previous experiments, we evaluated the stability over five contexts with five seeds. In this section, we consider a larger set of contexts. We consider one seed, which enables us to add nine additional contexts (14 contexts in total). We consider two models, Mistral-7B-Instruct-v0.2 and Mixtral-8x7B-Instruct-v0.1, as those were among the most stable models in all previous experiments.

**Fig 10:**
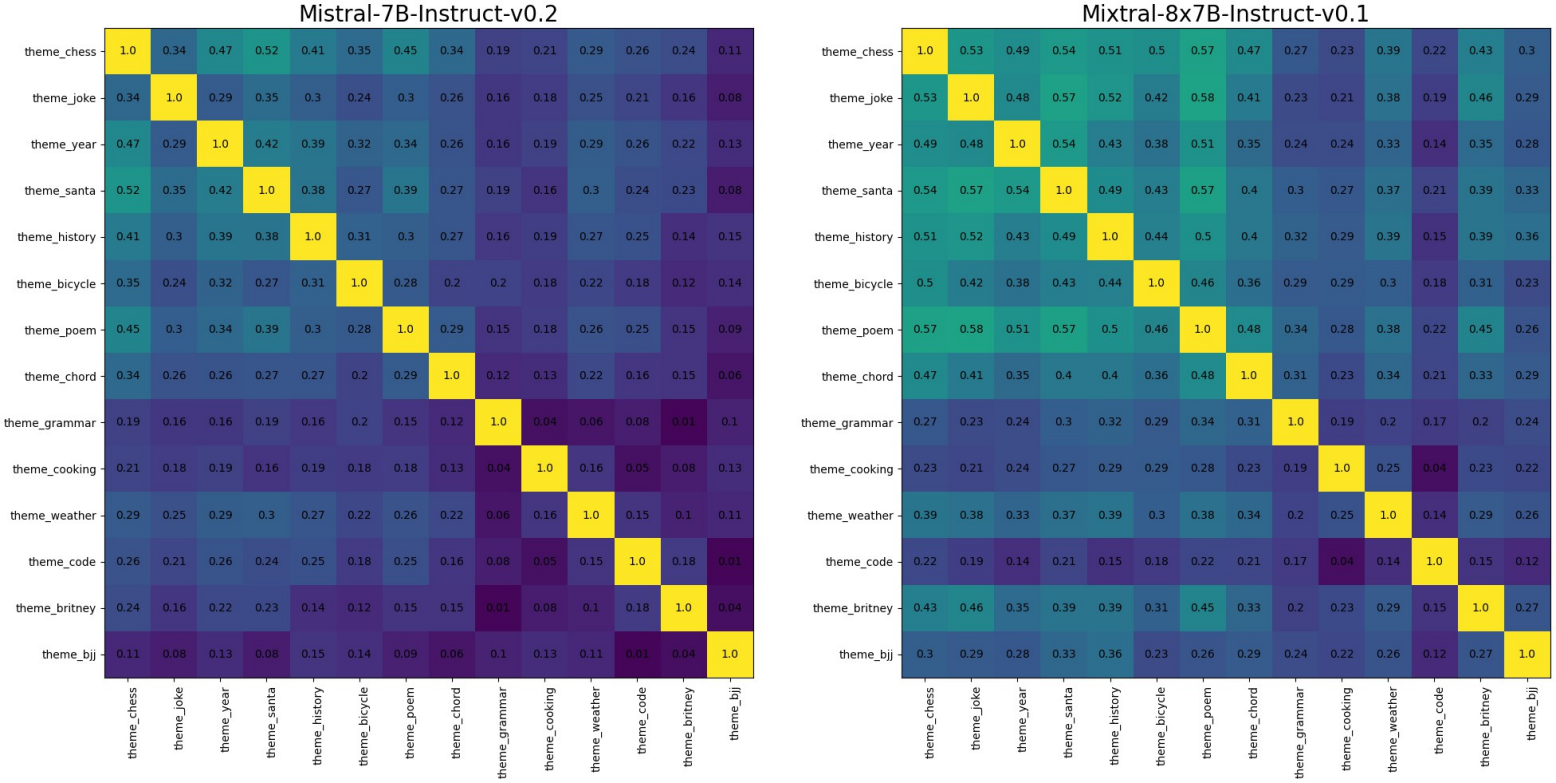
Rank-Order stability on additional contexts. Pair-wise Rank-Order stability of personal values (PVQ) exhibited by simulated fictional characters. The Mixtral-Instruct-8x7B-v0.1 model overall exhibited higher stability than Mistral-7B-Instruct-v0.2. For both models, lower stability is observed in longer contexts (bottom right corner).

Fig 10 shows stability between each pair of contexts for Mistral-7B-Instruct-v0.2 and Mixtral-8x7B-Instruct-v0.1. The average stability for those models is 0.215 and 0.334 respectively. Mistral-7B-Instruct-v0.2 exhibited lower stability in a majority of comparisons (except the topic of code). These results are consistent with those in previous experiments with five contexts (Fig 4). Furthermore, the contexts in Fig 10 are ordered based on the length of the initial message. We can see that longer contexts (bottom right) are characterized by lower stability (darker shades of purple). This suggests that context length plays a significant role on the stability of expressed values.

### What influences the model’s stability?

In this section, we will analyze the effect of various factors in the model’s stability. We consider: model size, the training mechanism, quantization, and the dataset size and content. First, we compare models within the same family to control for other, more complex factors which greatly vary between different families (e.g. data curation policy or instructions given to annotators). And then, we more generally analyze factors across different families. The following analysis will be made with respect to Rank-Order stability on PVQ (Fig 4, on downstream tasks (Fig 8), and with respect to Ipsative stability of PVQ (Fig 5).

#### Model size

In all our experiments, we observe a consistent trend of increasing stability with model size in the Qwen family. However, this is confounded by the increase in the training dataset size in those models. Furthermore, despite large differences in size, all LLaMa-2 models consistently exhibit low stability, with the exception of the Ipsative stability of LLaMa-2–70B-chat (Fig 5) and a modest Rank-Order stability on the Religion downstream task of both LLaMa-2–70B models (Fig 8C). Different Mistral models greatly vary in their stability despite their same size. Overall, despite higher stability being associated with larger models, no strong conclusions can be made with respect to model size.

#### Training mechanism

All models are first trained by supervised fine-tuning (SFT) to model a large corpus of text, i.e. base models. Those base model are often fine-tuned to follow instructions or for conversations, i.e. instruct or chat models. This can be done in by further fine-tuning: by SFT on an instruction on chat dataset, by DPO, or by RLHF. In the most complex setting, models also can be finetuned first with SFT and then with DPO or RLHF.

In our experiments, an effect of DPO fine-tuning was observed for the Mixtral-8x7B-v0.1 model in all experiments except the Stealing downstream task. The newer Mistral SFT instruction tuned model (Mistral-7B-Instruct-v0.2) is the most stable in the family and a large gap is observed with the repsect to the previous version (Mistral-7B-Instruc-v0.1) and with the base model (Mistral-7B-v0.1), with the DPO model (zephyr-7b-beta) in between. This suggests that simple SFT instruction tuning can be very powerfull when used with adequate training data. In the LLaMa-2 models, no effect was observed as a consequence of RLHF, except for the Ipsative stability and Rank-Order stability on the religion downstream task. Overall, the fine-tuning by DPO and SFT appear to be beneficial (provided adequate traning data), and no clear conclusions can be made for the benefit of using RLHF.

#### Quantization

Both models from the Mixtral family were evaluated with 16bit and 4bit precision. Across all experiments (Figs 4, 5, 8 and 9), we observe a slight but consistent drop in stability as a consequence of this quantization.

#### Dataset size and content

To analyze the effect of dataset size, we can consider the LLaMa-2 and Qwen model families. LLaMa-2 models were all trained with the same 2T token dataset, and, as discussed above, do not overall exhibit large changes in stability. The Qwen family exhibits a consistent trend of increasing stability with dataset size. This can also be due to the increase in model size, but given the unclear impact of model size in other families (as discussed above), we hypothesize that the dataset size is more important than model size for stability.

In addition to the dataset size, its content and quality are another important aspect. The dataset content reflects the provider’s policy used for collecting and filtering the dataset, as well as for instructing the annotators. The biggest impact of data content is observed in the Mistral family, where Mistral-7B-v0.1, Mistral-7B-Instruct-v0.1, and Mistral-7B-Instruct-v0.2 models of the same size were trained by SFT on different datasets. These three models greatly differ in terms of stability, either due to the different dataset quality or due to dataset size (which is not disclosed). Similarly, we can compare models from the GPT-3.5 family, for which no details were released. The two models were released in January 2024 (gpt-3.5-turbo-0125) and in November 2023 (gpt-3.5-turbo-1106). The newer version was likely made to be more aligned with the OpenAI’s policy, partially through fine-tuning on new data. In all our experiments, we observe a slight, but very consistent drop in stability from the older to the newer model. We hypothesize that this is due to alignment fine-tuning, which could prevent the model from accurately simulating controversial historical figures or evil fictional characters, and also make the model align itself more to the current interlocutor and situation (e.g. by agreeing with the user [29]). Overall, the dataset has a large effect on the model stability, which can be increased with a bigger, higher quality dataset. However, depending on the design choices made by the model provider, higher quality dataset can also decrease stabilty if the goal is to make a model more “aligned” with a single value profile.

In comparing models across different families, the minimal model size to exhibit some stability (*r* > 0.3) is 7B parameters (Mistral-7B-Instruct-v0.2), and the minimal dataset size 3T tokens (Qwen-72B). Datasets used by the Mistral company seem to be beneficial for stability, as evidenced by the higher stability exhibited by smaller models (7B and 46.7B) compared to other families. We hypothesize, that the LLaMa-2 models’ lower stability is due to the smaller dataset size (2T tokens), and the lower stability of GPT-3.5 model due to the “alignment” fine-tuning.

## Conclusion

This paper presents the first study into the stability of values expressed by Large Language Models. We consider (interpersonal) Rank-Order stability and (intrapersonal) Ipsative stability. We evaluate value stability over different contexts induced by simulating conversations about different topics. We conduct experiments with and without instructing the models to simulate particular personas. Over our experiments, we observed consistent trends of value stability: Mixtral, Mistral, GPT-3.5 and Qwen model families were more stable. These trends are also confirmed on downstream behavioral tasks. LLMs instructed to simulate personas exhibit much lower than human stability (despite the comparison being skewed in their favor), which further diminishes over longer conversations. This insight highlights the limitation of the studied LLMs and motivates future research on models specialized in simulating coherent populations of individuals.

This paper highlights how seemingly unrelated context changes can result in unpredictable and unwanted changes in behavior. We argue that context-dependence, and more precisely, value stability, should be seen as another dimension of LLM comparison alongside knowledge, model size, speed, and similar. Instead of evaluating LLMs with many different questions from a single minimal context, they should also be evaluated (in terms of their context-dependence and value stability) with the same questions asked in many different contexts. This study presents a first step in that direction.

### Limitations

Due to computational requirements for evaluating LLMs, most of our experiments consider only five different conversation topics and rather short conversations. Greatly increasing the number of topics and conversation length could provide more precise insights into the stability of various models. For one model, Rank-Order value stability is estimated from 50/60k queries and Ipsative stability from 10/12k queries (depending on the simulated population). For reference, MMLU [31] (a commonly used general knowledge benchmark) contains 14k test questions.

Given that most LLMs have primarily been trained on English text, we present contexts and the questionnaire in English as well. Repeating the study in different languages would contribute to understanding the cultural biases in LLMs.

This paper studied one of the issues with a common practice of directly applying psychological questionnaires to LLMs: the extreme context dependence, which is higher than what one might expect in humans. However, the question under which conditions can different questionnaires be applied to LLM still remains largely open. It is possible that other aspects, in addition to context-dependence, need to be addresed to make stronger claims about the value expression in LLMs.

### Future work

We believe that this paper opens many research avenues regarding context-dependence and value stability of LLMs. Similar questions to those explored in this paper could be explored for personality traits, cultural values, cognitive abilities and knowledge. An interesting direction is to explore if the same model can exhibit high stability in both settings with and without the persona instruction, or if specialized models are required. Increasing the LLMs’ interpretability could help understand how to increase their stability. This paper opens a new area of research in creating, evaluating and analyzing models specialized in simulating coherent and diverse populations. Such models are needed for many applications such as replicating human studies [7], simulating social interactions [44], training teachers [26], and many more.

## Supporting information

S1 Appendix Additional details on the methods. (PDF)

S2 Appendix Additional experiments and analyses. (PDF)

S1 Fig Statistical comparison of models’ Rank-Order value stability for LLMs simulating fictional characters. This accompanies results shown in Fig 4A. Black cells denote statistically significant difference between models.
(SVG)

S2 Fig Statistical comparison of models’ Rank-Order value stability for LLMs simulating real-world personas. This accompanies results shown in Fig 4B. Black cells denote statistically significant difference between models.
(SVG)

S3 Fig Statistical comparison of models’ Ipsative value stability for LLMs without the persona setting instructions. This accompanies results shown in Fig 5. Black cells denote statistically significant difference between models.
(SVG)

S4 Fig Statistical comparison of models’ Rank-Order value stability on the downstream Donation task. This accompanies results shown in Fig 8A. Black cells denote statistically significant difference between models.
(SVG)

S5 Fig Statistical comparison of models’ Rank-Order value stability on the downstream Stealing task. This accompanies results shown in Fig 8B. Black cells denote statistically significant difference between models.
(SVG)

S6 Fig Statistical comparison of models’ Rank-Order value stability on the downstream Religion task. This accompanies results shown in Fig 8C. Black cells denote statistically significant difference between models.
(SVG)
