# Supplementary material for: Stick to your role! Stability of personal values expressed in large language models
Source: PLoS One. 2024 Aug 26;19(8):e0309114. doi: 10.1371/journal.pone.0309114 (PMC11346639; doi:10.1371/journal.pone.0309114)
Supplement: S2 Appendix — (PDF) [file pone.0309114.s002.pdf]

1 Additional experiments and analyses

1.1 Visualization of Mixtral-Instruct value expression

In this section, we visualize the PVQ values expressed by the Mixtral-Instruct model along different contexts and seeds. We use PCA [1] to visualize 250 dimensions (5 seeds x 5 contexts x 10 values) dimensions as two PCA components with $R^2 = 0.29$ and $R^2 = 0.12$ explained variance ratios. We used GPT-4 to classify characters into positive, neutral (more complex), and negative using the following prompt:

```
Classify the following characters into positive/neutral/negative.

Create a two column table with the first column being the name of
the character and the second being the classification.

Gandalf
...
Gimli
```

Fig. 1 shows the representation of 60 fictional characters classified into positive, neutral (more complex) and negative characters. We can see that positive characters are grouped on the left, negative characters on the right, and neutral in the middle. This shows the Mixtral-Instruct model expressed values in a semantically plausible way.

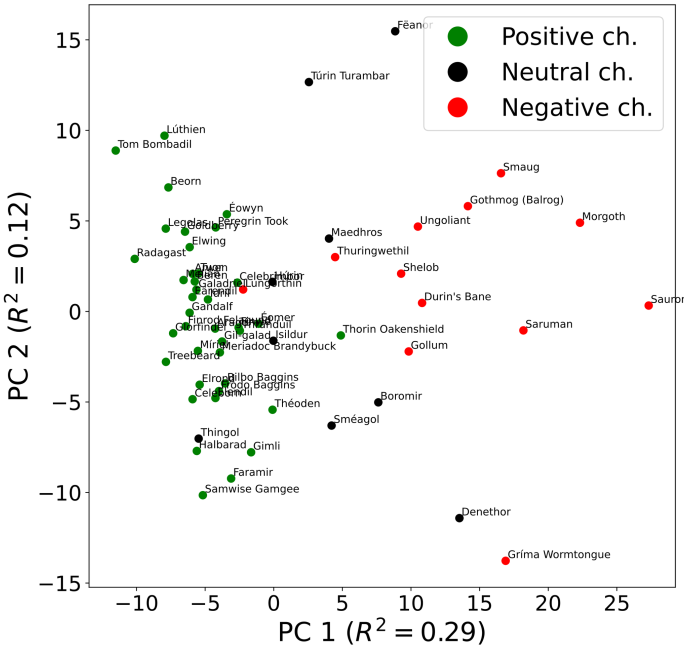

Fig 1. PCA representation of different fictional characters simulated by the Mixtral-Instruct model. Positive characters (green) are grouped on the left side, negative characters (red) on the right side, and neutral (more complex) characters are in the middle.

1.2 Do simulated personas' value profiles approach a *neutral* value profile with longer conversations?

.

In the main text, we studied how stability changes as conversations get longer. For the Mixtral-Instruct (that was instructed to simulate fictional characters), we observed that Rank-order stability diminished and Ipsative stability stayed the same with longer conversations. This implied that simulated personas' value profiles moved away from the instructed personas towards some neutral value profile. Here, we experimentally confirm this hypothesis by estimating the distance of simulated personas' value profiles to an estimated neutral value profile. The distance is computed as Ipsative stability: correlation between the order of values in a simulated individual to those of the neutral value profile.

The neutral profile is estimated as follows. We evaluate the Mixtral-Instruct model without the persona setting instructions and without simulating a conversation (i.e. the questionnaire queries are given straight away). We repeat this process with 50 permutations in the order of suggested answers. To estimate the neutral profile, we average the value ranks over those permutations as shown in the following pseudocode:

```
# value_score.shape == (50, 10)
value_scores = evaluate_model("Mixtral-Instruct", n_perm=50)
all_ranks = []
for i in range(50):
    # permutation_value_profile.shape == (10)
    permutation_scores = value_scores[i]
    permuataion_value_ranks = compute_ranks(permuatation_scores)
    all_ranks.append(permutation_values_ranks)

# all_ranks.shape == (50, 10)
# neutral_value_profile.shape == (10)
neutral_value_profile = all_rank.mean(axis=0)
```

Fig. 2 shows the similarity of simulated personas' value profiles to the neutral profile (blue), with the Rank-Order stability of simulated individuals between contexts (black). We can see that, as Rank-Order stability diminishes, simulated personas' value profiles move closer to the default profile. This confirms our hypothesis that the diminishing Rank-Order stability is due to the model gradually "ignoring" the persona inducing instruction and moving all simulated personas' value profiles closer to the neutral one.

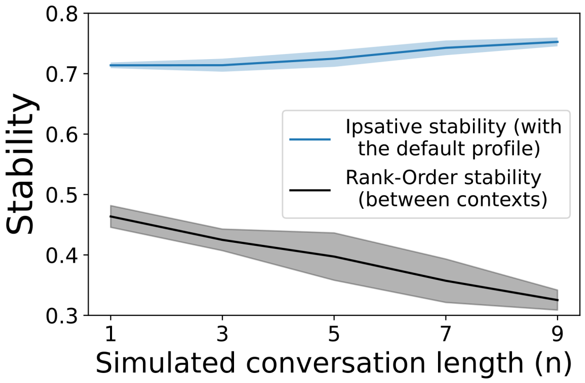

Fig 2. Similarity of Mixtral-Instruct simulated fictional characters' value profiles with the neutral value profile (blue) compared to the Rank-order stability. As conversations gets longer, simulated value profiles move away from the instructed persona toward a neutral one, resulting in lower Rank-order stability.

1.3 Does the order of simulated participants move away from the *neutral* order as conversations get longer

We study how the order of simulated participants moves away from the *neutral* participant order. The *neutral* participant order is estimated by instructing the model (Mixtral-Instruct) to simulate personas (fictional characters) but without simulating conversations, i.e. the questionnaire is given directly after the instruction. We compute two types of Rank-Order stability: stability between contexts (as in the main text) and stability with respect to the *neutral* order.

Stability between contexts is computed with the following equation:

$$RO_{cont} = \langle \text{corr}(c_1, c_2) \rangle_{c_1, c_2 \in C, c_1 \neq c_2}$$

,RO_{cont} is the stability between contexts, C is a set of participants orders in different contexts, and *corr* computes the correlation.

Stability with respect to the *neutral* order is computed with the following equation:

$$RO_{neut} = \langle \text{corr}(c_1, n) \rangle_{c_1 \in C}$$

, where RO_{net} is the stability w.r.t. the neutral order, C is a set of participants orders in different contexts, n is the *neutral* participant order, and *corr* computes the correlation. Both types of Rank-Order stability are computed with five seeds and averaged.

Figure 3 shows the stability between contexts (black) and the stability w.r.t. the *neutral* order (blue). Both stability measures diminish as conversations get longer. This implies that the orders of simulated participants are moving away both from the *neutral* order and from each other. Stability w.r.t. the *neutral* order is consistently higher than the stability between contexts. This implies that the *neutral* order is in between the order in different contexts, i.e. simulated conversations are pulling the participant orders in different directions away from the *neutral* order.

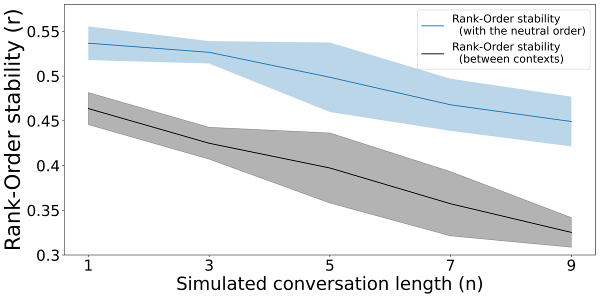

Fig 3. Rank-Order stability between different contexts (simulated conversations) and with respect to the *neutral* order of participants (without simulating a conversation). As simulated conversations get longer, participant orders move away (become more different) from both the *neutral* order and each other. The *neutral* order is in between the per-context orders (as the stability w.r.t. the *neutral* order is higher than stability between contexts)

1.4 Is the LLaMa-2 models' lower stability caused by the used persona induction method ?

In the main text, LLaMa-2 chat models exhibited very low stability, but those models are also the only ones (apart from zephyr-7b-beta) which used the prompt template with the *system message* input. Furthermore, Mistral-Instruct and Mixtral-Instruct, which showed high stability, used the template without the *system message* input.

Therefore, we found it relevant to check that LLaMa-2 low stability is not caused by the prompting template but by the model itself.

Figure 4 shows the three LLaMa-2 chat tuned models with the two prompting templates. It compares inducing the persona through the *system message* (denoted by "*_sys"), as was done in the main text, to inducing it through the *user message* (denoted by "*_no_sys"), as was done for other models. We can see that neither prompt template enables the LLaMa-2 chat models to exhibit higher stability. This implies that the exhibited low stability is due to the models themselves, and not merely due to the choice of a prompting template.

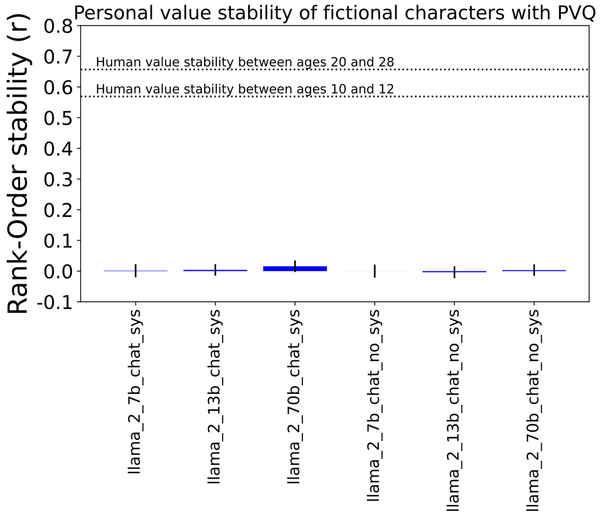

Fig 4. Rank-order value stability ($Mean \pm SI(\alpha = 0.05)$) of chat-tuned LLaMa-2 models when the persona is induces through the *system message* (as was done in the main text) compared to the *user message* input (as was done for other models). LLaMa-2 models do not exhibit value stability in either setting. This implies that the low stability is due to the LLaMa models themselves, and not due to the choice of a prompting template.

References

1. S KPFR. LIII. On lines and planes of closest fit to systems of points in space. The London, Edinburgh, and Dublin Philosophical Magazine and Journal of Science. 1901;2(11):559–572. doi:10.1080/14786440109462720.
